# No obvious genetic erosion, but evident relict status at the westernmost range edge of the Pontic‐Pannonian steppe plant *Linum flavum* L. (Linaceae) in Central Europe

**DOI:** 10.1002/ece3.2990

**Published:** 2017-07-14

**Authors:** Kristina Plenk, Katharina Bardy, Maria Höhn, Mike Thiv, Matthias Kropf

**Affiliations:** ^1^ Institute for Integrative Nature Conservation Research University of Natural Resources and Life Sciences (BOKU) Vienna Austria; ^2^ Department of Botany and Botanical Garden of Soroksár Faculty of Horticultural Science Szent István University Budapest Hungary; ^3^ Botany Department Stuttgart State Museum of Natural History Stuttgart Germany

**Keywords:** AFLPs, cpDNA sequence variation, genetic diversity, glacial relict populations, peripheral transect, phylogeography

## Abstract

We investigate patterns of genetic variation along an east–west transect of Central European populations of *Linum flavum* and interpret the Quaternary history of its peripheral populations, especially those at the westernmost isolated range edge, discussing their migrations and possible relict status. We defined our peripheral transect across three study regions from Central Hungary, eastern Austria to southwestern Germany. Using AFLP fingerprinting and cpDNA sequence variation (*rpL16* intron, *atpI‐H*), we analyzed 267 and 95 individuals, respectively, representing each study region by four populations. Hierarchical AMOVA (AFLPs) indicated significant variation among study regions (12% of total variance) and moderate differentiation between populations (10%). Population differentiation was high at the westernmost range edge (11.5%, Germany), but also in the east (13.4%, Hungary), compared to the Austrian study region (8.6%). Correspondingly, AFLP diversity was highest in the center of the study transect in eastern Austria. CpDNA haplotypes support a pattern of regional structuring with the strongest separation of the westernmost range edge, and some haplotype sharing among Austrian and Hungarian individuals. Equilibrating nucleotide versus haplotype diversity patterns, the highly diverse populations at the Pannonian range edge (Austria) indicate long‐term persistence, while Central Pannonian populations are obviously effected by recent bottlenecks. Intermediate nucleotide, but high haplotype diversity within the westernmost exclave (Swabian Alb), is indicative of a founder bottleneck during its pre‐LGM or early postglacial migration history, followed by sufficient time to accumulate cpDNA variation. The not obviously reduced genetic diversity and distinctiveness of *L. flavum* at the westernmost range edge suggest a long‐term persistence (relict status) of populations in this region, where the species has survived probably even the Würm glaciation in extra‐Mediterranean refugia. This genetic relict variation represents an important part of the overall genetic diversity found in the western periphery of this steppe plant and highlights the high conservation priority of respective gene pools.

## 1 INTRODUCTION

Glacial and interglacial periods of the Quaternary led to several drastic range shifts of European organisms during the last ca. 2.5 million years, with climatic oscillations being most pronounced starting 800,000 years ago (ya; Lowe & Walker, 2014). Induced by climate warming in the early postglacial period (Gliemeroth, 1996; Lang, 1994) or possibly even in pre‐LGM times (cf. Wilmanns, 2005), more thermophilic steppe species began to expand from Pontic, Central Asian and sub‐Mediterranean areas toward the (north)west (e.g., Central Europe). The further increasing temperature as well as the (coincident) extinction of large herbivores and forest fires destroying pine forests in the early Holocene enabled the expansion of broad‐leaf forests mainly from their more southern glacial refugia (Feurdean et al., 2012; Johnson, 2009; Sümegi, 1999; Willis, Braun, Sümegi, & Tóth, 1997). Hence, steppelike grasslands in Central Europe were again restricted to areas where edaphic and climatic conditions excluded dense tree cover (Bredenkamp, Spada, & Kazmierczak, 2002; Feurdean et al., 2015; Kuneš et al., 2015; Magyari et al., 2010). However, besides these large‐scale postglacial expansions and contractions across vast areas of Central Europe, also an earlier arrival and/or (more small‐scale) survival of thermophilic species during the Würm glaciation (i.e., LGM, ca. 20,000 ya), not only in Mediterranean refugial core areas, but further north (in the Carpathian region or even north of the Alps, e.g., in southwestern Germany) in extra‐Mediterranean refugia, has been hypothesized (cf. Kajtoch et al.*,* 2016; Schmitt & Varga, 2012; Stewart & Lister, 2001).

Originating from both (northern) relict and (southern) refugial localities, steppelike grasslands again expanded when humans started clearing forests and using land for grazing livestock and farming (Chapman, Magyari, & Gaydarska, 2009; Magyari, Chapman, Fairbairn, Francis, & de Gunzman, 2012; Magyari et al., 2010; Willis, Sümegi, Braun, Bennett, & Tóth, 1998). These suitable conditions were mostly present until the late 19th century before the intensification of agricultural land use resulted in a tremendous decline of (managed) open, steppelike habitats. However, remnants of these habitats could still be found in the mid‐20th century in more remote areas (e.g., the Swabian Alb). Today, the remaining steppe areas are restricted to sites unsuitable for intensive farming (e.g., dry calcareous or sandy grasslands, rocky slopes, gravel, loess or salt soils, and patches in open xerophilous forest communities), leading to fragmented and isolated populations of therefore today often endangered steppe species (Meindl, 2011; Poschlod & WallisDeVries, 2002). Based on this general scenario of steppe vegetation history, thermophilic (nonwoody steppe) species arrived in Central Europe during more than one westward expansion waves in pre‐LGM, glacial, or early postglacial times and could have also persisted in situ within extreme habitats described above throughout the Holocene (i.e., without interim retreat, indicating their possible relict status; cf. Stewart & Lister, 2001; Schmitt & Varga, 2012; Kajtoch et al.*,* 2016; but see also Wilmanns, 2005).

Studies on the relict status of (Central) European species, addressing the phylogeography and Quaternary history not only of steppe plants, controversially discuss the (genetic) constitution of so‐called old rare species (e.g., Durka et al., 2013; Hensen et al., 2010; Pérez‐Collazos, Sanchez‐Gómez, Jiménez, & Catalán, 2009; Šmídová, Münzbergová, & Plačková, 2011; Vogler & Reisch, 2013). Such “old” relict populations are not necessarily characterized by small population size or reduced fitness, but by comparatively high genetic diversity (e.g., due to long‐term in situ survival in extra‐Mediterranean refugia, cf. Schmitt & Varga, 2012) and genetic distinctiveness (e.g., due to isolation, genetic drift and/or local adaptation, see also review by Kajtoch et al., 2016). Otherwise, different reviews addressed biogeographical hypotheses on species’ distribution ranges and discussed the characteristics of peripheral populations (i.e., low genetic diversity, small population sizes, increasing population differentiation, reduced gene flow, and subsequently reduced fitness), partly controversially (Abeli, Gentili, Mondoni, Orsenigo, & Rossi, 2014; Eckert, Samis, & Lougheed, 2008; Kajtoch et al., 2016; Sagarin & Gaines, 2002; Sexton, McIntyre, Angert, & Rice, 2009). Furthermore, some of the mentioned characteristics (e.g., low genetic variation and small population sizes due to founder effects) of isolated range‐edge populations might also give evidence for (recent) long‐distance dispersal events, but source and founder populations are expected to be detectable due to their (high) genetic similarity (cf. Wróblewska, 2008).

**Figure 1:**
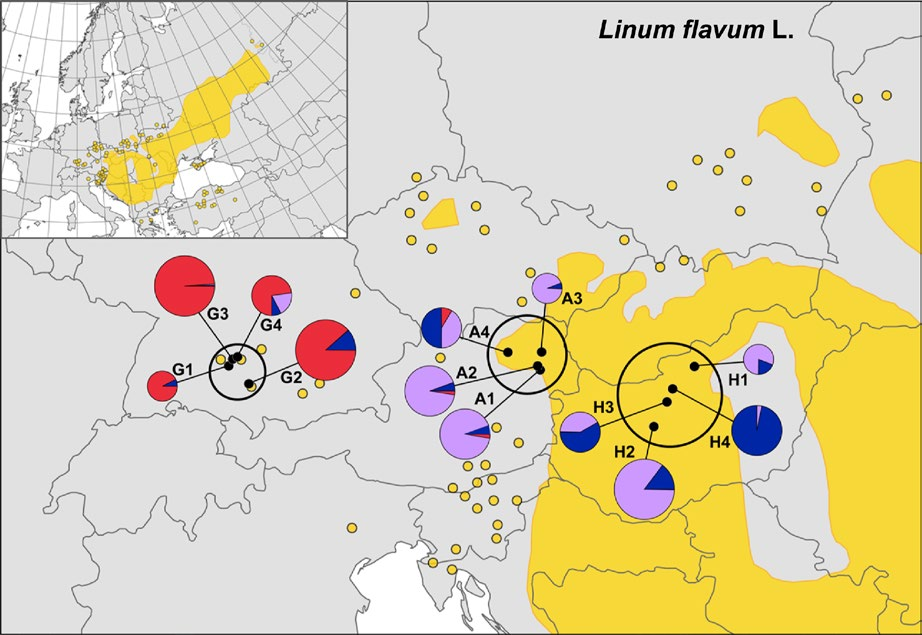
Distribution and sampling of *Linum flavum*. Distribution map of *Linum flavum* L. based on Meusel et al. (1978); continuous distribution represented as full‐colored parts, disjunct occurrences are shown as colored points. The inset shows the entire distribution range of the species. Within our peripheral transect, the three study regions Germany, Austria, and Hungary are marked with black circles; sampled populations are represented by black dots. Pie charts show AFLP‐based proportions of admixture based on *K* = 3 from the Bayesian mixture analysis (size of the pie charts refers to the population size categories). For population abbreviations and population sizes, see Table 1

In this study, we investigate the peripheral occurrences of the perennial steppe plant *Linum flavum* (Linaceae), which can be found in the sub‐Mediterranean, Balkan, Pontic, Pannonian, and East European regions (Meusel, Jäger, Rauschert, & Weinert, 1978; cf. Figure 1), reaching its westernmost distribution limit in southern Germany. In a recent study, Cieślak (2014) investigated the phylogeographic history of *L. flavum* and other Pontic‐Pannonian species, using AFLP fingerprinting with a strong focus on Central Europe. However, the latter work did not consider the westernmost occurrences of the species and represented the southeastern Pannonian region (i.e., Austria and Hungary) with just two study populations. Utilizing a Central European peripheral study transect, we include the Central Hungarian region (i.e., the Pannonicum, see Figure 1), the western Pannonian range edge in eastern Austria and the westernmost exclaves in southern Germany (namely the Swabian Alb in Baden‐Wuerttemberg and one population from the Bavarian Iller river valley, i.e., only 40 km apart; subsequently treated as “Swabian Alb,” jointly) in our study. Hypothesizing a long‐term persistence within the latter region at sites that are still populated today (Wilmanns, 2005), we assume that the species has arrived at the Swabian Alb during a pre‐LGM or an early (first) postglacial expansion wave of steppe elements toward Central Europe and persisted there throughout the Holocene, therefore representing relict populations today. In the case of such a long‐term persistence, we expect to identify comparatively high or at least equally high genetic within‐population diversity compared to the more eastern (Pannonian) habitats and the development of a unique gene pool in the westernmost Central European exclaves. In this respect, we will also try to detect signs of selective versus nonselective genetic variation within the latter gene pool. An alternative scenario represents a (more) recent colonization during a younger expansion wave (e.g., supported by the anthropogenic land opening) or a long‐distance dispersal (LDD) event. In these latter two cases, we would predict low genetic diversity in the Swabian Alb exclave and also less genetic differentiation among regions (but also among populations). Reflecting a (very) recent LDD event, a high genetic similarity might indicate the close relationship between specific source and founder populations among regions.

Based on these considerations, we are aiming at (1) the evaluation of the distribution of genetic variation and diversity along our peripheral study transect, and (2) testing our hypotheses on the relict status of the Swabian Alb populations and reconstructing the Quaternary history of *L. flavum* at its westernmost range edge in Central Europe by applying two different molecular marker systems, that is, amplified fragment length polymorphisms (AFLPs) and chloroplast DNA sequence analyses. Consequently, we will answer the question whether the specific biogeographical history of this species at the (westernmost) range edge covers basically expected patterns regarding peripheral populations (i.e., showing genetic erosion and strong population differentiation within the westernmost Swabian Alb exclave).

## 2 MATERIALS AND METHODS

### 2.1 The study species *Linum flavum* L.

*Linum flavum* L. belongs to the evidently monophyletic and predominantly yellow‐flowering group, sect. *Syllinum* Griseb., which is widespread in the northern hemisphere and evolved about 19–17 mya ago (McDill, Repplinger, Simpson, & Kadereit, 2009; Repplinger, 2009).

*Linum flavum* typically can be found in dry grasslands and slopes, along woodland margins or in dry pine forests (Ockendon & Walters, 1968); but less frequently the species populates also mesic sites. This perennial, calcicole, and thermophilic species reaches a height of about 50 cm with conspicuous branched inflorescences. The deep yellow flowers blossom from June to late July (Demuth et al., 1992; Fischer, Oswald, & Adler, 2008; Oberdorfer, 2001). The species is described as predominantly outcrossing (but see Repplinger, 2009). As pollinators, bees (e.g., *Osmia mocsaryi*, a wild bee which uses the petals of *L. flavum* to tapestry their nesting holes; Demuth et al., 1992), bumblebees, wasps, bombylides, and syrphids have been recorded. Seeds of *L. flavum* are self‐spreading or partially dispersed by wind. However, Münzbergová (2004, 2013) described this species as overall dispersal‐limited. Chromosome numbers of the diploid species are documented as 2n = 30 (Oberdorfer, 2001) and 2n = 32 (Dobeš & Vitek, 2000).

In Hungary, *L. flavum* is documented as “near threatened,” which describes a lower risk level for becoming endangered in future, but not a current “threat”‐category. As the species is getting rarer in a westward direction, the conservation status is declared as “vulnerable” (or regionally “endangered”) in Austria and generally “endangered” in Germany (Király, 2007; Korneck, Schnittler, & Vollmer, 1996; Niklfeld & Schratt‐Ehrendorfer, 1999).

### 2.2 Study transect and sampling design

In the Pannonian region, steppe vegetation is restricted to sites with well‐drained soils or soils characterized by high salt concentrations (i.e., physiologically “dry” soils) in combination with low precipitation rates (<600 mm per year), for example, the Hungarian Plains in Central Hungary (Fekete, Molnár, Magyari, Somodi, & Varga, 2014; Soó, 1959) or the Vienna basin in Eastern Austria (Niklfeld, 1964). However, also far west of the border of the Pannonian Region, few isolated exclaves of xerothermophilic species occur in western and southern Germany, where <1,000 ha represent the total steppelike vegetation (e.g., at the Swabian and Franconian Alb, the Danube valley, the Main valley, the Nahe valley, as well as the Upper and Middle Rhine valley; Meindl, 2011).

Our Central European study transect, where only *L. flavum* s.str. is known (Bettinger et al., 2013; Fischer et al., 2008), represents a section of this peripheral distribution area, covering three different study regions: (1) Central Hungary, located within the more continuous distribution area of the study species (Meusel et al., 1978); (2) the Pannonian region of Eastern Austria, at the border of this continuous Pannonian occurrence; and (3) southern Germany, representing the westernmost exclave at the absolute edge of the species’ distribution. Within the latter region, *L*. *flavum* reaches its distribution limit in Baden‐Württemberg (Swabian Alb) where it only occurs in few, mostly scattered and isolated populations.

We randomly collected leaf material from four populations per study region and 24 individuals per population (Table 1). The minimum distances between two populations within a study region were 8.1 km (Hellebarten–Klingenstein; Swabian Alb), 7.0 km (Eichkogel–Perchtoldsdorf; Lower Austria), and 21.9 km (Erd–Üröm; Central Hungary). The number of flowering individuals per population was estimated to determine a population size category for each population sampled.

**Table 1:** Distribution of genetic diversity across study populations

Code	Country	Sampling location	Altitude m a.s.l.	Coordinates (E, N)	Estimated Pop. size categories	N AFLP original/final	PLP	*H*_E_	DW	N cpDNA original/final	Number of haplotypes	Nucleotide diversity (π)	Haplotype diversity (*h*)
LfG1	Germany	Büchelesberg	540	09°44′; 48°18′	1	24/24	0.32	0.08	73.91	9/6	5	0.0009 ± 0.0007	0.933 ± 0.122
LfG2	Germany	Heimertingen	560	10°09′; 48°03′	4	24/20	0.34	0.09	67.76	9/8	7	0.0013 ± 0.0009	0.964 ± 0.077
LfG3	Germany	Hellebarten	550	09°49′; 48°24′	4	24/22	0.29	0.08	54.06	9/9	7	0.0009 ± 0.0006	0.833 ± 0.127
LfG4	Germany	Klingenstein	500	09°55′; 48°26′	2	24/19	0.28	0.08	51.13	8/8	4	0.0007 ± 0.0005	0.750 ± 0.139
LfA1	Austria	Eichkogel	315	16°17′; 48°04′	3	24/23	0.40	0.11	66.49	9/9	6	0.0013 ± 0.0009	0.833 ± 0.127
LfA2	Austria	Perchtoldsdorfer Heide	330	16°15′; 48°07′	3	24/22	0.42	0.11	74.59	9/8	8	0.0014 ± 0.0009	1.000 ± 0.063
LfA3	Austria	Bisamberg	300	16°22′; 48°19′	1	24/23	0.51	0.14	151.67	9/9	6	0.0010 ± 0.0007	0.917 ± 0.073
LfA4	Austria	Höbenbach	350	15°39′; 48°21′	2	24/23	0.38	0.10	71.99	8/7	4	0.0008 ± 0.0006	0.810 ± 0.130
LfH1	Hungary	Buják	280	19°32′; 47°52′	1	24/20	0.33	0.09	68.74	9/9	5	0.0009 ± 0.0006	0.722 ± 0.159
LfH2	Hungary	Belsöbarand	120	18°32′; 47°06′	4	24/24	0.37	0.09	68.88	9/8	7	0.0016 ± 0.0010	0.964 ± 0.077
LfH3	Hungary	Erd	280	18°52′; 47°26′	2	24/24	0.35	0.09	65.41	8/8	5	0.0010 ± 0.0007	0.893 ± 0.086
LfH4	Hungary	Üröm	192	19°01′; 47°35′	3	24/23	0.27	0.07	62.72	8/6	2	0.0002 ± 0.0003	0.503 ± 0.172

Lf, *Linum flavum* L.; G, Germany; A, Austria; H, Hungary; PLP, proportion of polymorphic fragments; *H*_E_, Nei's gene diversity; DW, frequency down‐weighed marker values (calculated from sums).

Population size categories: 1 = < 100; 2 = 100–200; 3 = 200–500; 4 = 500–1000 individuals.

Sampling information, population code, category of estimated population size, estimates of genetic diversity based on AFLP/cpDNA data and number of haplotypes of twelve *Linum flavum* L. populations sampled in the study regions Swabian Alb (Germany), Lower Austria, and Central Hungary.

Isolation of total genomic DNA from silica dried leaf material (ca. 20 mg) was carried out using sterilized glass pellets for grinding and the DNeasy™ Plant Mini Extraction Kit (QIAGEN, Hilden, Germany) according to the manufacturer's protocol.

### 2.3 AFLP fingerprinting

AFLP profiles of 288 individuals (Table 1) were generated based on the original protocol of Vos et al. (1995) with some modifications described by Kropf, Comes, and Kadereit (2006) and Kropf (2012).

Digestion of genomic DNA was carried out with the restriction enzymes *Mse*I and *Eco*RI. Preselective amplifications were performed with primer pairs containing each one selective nucleotide. For selective amplifications, three primer combinations (E+ACG/M+CGG [E37/M57], E+AGA/M+CTG [E39/M61], E+ATG/M+CGG [E45/M57]; Kropf, Kadereit, & Comes, 2003; Kropf et al., 2006) were used with each two additional selective nucleotides and labeled by different fluorescence dyes (E37: NED™, E39: 6‐FAM™, E45: HEX™). The fluorescence labeled products were merged together with an internal size standard (ROX™, ET550‐R) and loaded on a MegaBACE DNA Analysis System with 48 capillaries (Amersham Biosciences, Freiburg, Germany). To receive a presence/absence matrix of AFLP fragments, the raw data were aligned with the internal size standard using the MegaBACE program Fragment Profiler 1.2 (Amersham Biosciences) and a peak height threshold of 50 relative fluorescent units (RFUs) within a range of 60–550 base pairs fragment length. A visual check of this matrix was performed to exclude misinterpretations (Meudt & Clarke, 2007).

To determine the reproducibility of fragments, six replicated individuals per plate (i.e., 41 replicated individuals in total) were included during lab work (Bonin et al., 2004; Meudt & Clarke, 2007). With those replicates an evaluation of the relative proportion of mismatches (0 vs. 1) compared to matches (1 vs. 1) across all AFLP profiles could be calculated (Knowles & Richards, 2005; Pompanon, Bonin, Bellemain, & Taberlet, 2005). Additionally, blind samples were included to check the runs for impurities (Bonin et al., 2004).

### 2.4 cpDNA sequencing

Sequences derived from the chloroplast were generated for 104 individuals representing the three study regions with four populations each. Within the plastid genome two regions were sequenced from eight to nine individuals per population: *rpL16 intron* (primers: *rpL16F71*,* rpL16R1516*; Shaw et al., 2005) and *atpI‐H* (primers: *atpI*,* atpH*; Shaw, Lickey, Schilling, & Small, 2007). Both have previously been tested for our study species (other cpDNA markers tested: *rpL32*,* rpS16, trnL‐trnF*). For the amplification step a 10 μl reaction mix (containing 5 μl Multiplex PCR Master Mix (QIAGEN), 2.2 μl H_2_O (Dnase/Rnase‐free; Life technologies, Carlsbad, NM, USA), 0.4 μl forward primer (Eurofins MWG Operon, Ebersberg, Germany), 0.4 μl reverse primer (Eurofins) and 2 μl of diluted DNA) run under the following conditions: initial 15 min at 95°C, followed by 35 cycles each of 45 s at 95°C, 45 s at 55°C, and 1 min at 72°C, then finally 10 min at 72°C. 12 μl of diluted PCR products were then sent to LGC Genomics (Berlin, Germany) for cycle sequencing using the same forward primers. Sequences obtained were manually aligned with BioEdit (version 7.2.5; Hall, 1999).

### 2.5 Data analyses

#### 2.5.1 AFLPs

A *genetic distance* analysis based on the complementary value of Nei and Li's similarity coefficient (Nei & Li, 1979) was performed with TreeCon (version 1.3b; Van de Peer & De Wachter, 1994; http://bioinformatics.psb.ugent.be/software/details/Treecon). The visualization of the distance matrix resulted in an unrooted neighbor‐net phenogram using SplitsTree4 (version 4.11.3; Huson & Bryant, 2006; http://www.splitstree.org/).

To determine *genetic structure*, we used Bayesian clustering running BAPS (version 3.2.; Corander, Waldmann, & Sillanpää, 2003; http://www.helsinki.fi/bsg/software/BAPS/) to reveal the optimal number of clusters (*K*) by the means of stochastic optimization. Clustering of individuals was carried out with a maximal number of 2–200 groups (*K*) and performing ten replicates. A Bayesian admixture analysis (Corander & Marttinen, 2006; based on the mixture clustering) was performed to investigate individual amount of admixture using 200 iterations for estimation of the admixture coefficient and 50 reference individuals from each population with 10 iterations each.

*Analyses of molecular variance* (AMOVAs) were calculated with ARLEQUIN (version 3.5.1.2; Excoffier & Lischer, 2010; http://cmpg.unibe.ch/software/arlequin35/Arl35Downloads.html). With different hierarchical approaches the differentiation among and within study regions or groups of populations was investigated using significance tests on the basis of 1,000 permutations. For testing *isolation‐by‐distance* (IBD) patterns (Hutchinson & Templeton, 1999; Wright, 1943), pairwise population *F*_ST_ values (Wright, 1951) were regressed on the geographical distance (km) between populations. The one‐tailed significance of the regression slope was evaluated by comparing the observed normalized Mantel statistic Z (Mantel, 1967) with its random distribution, obtained based on 9,999 permutations running NTSYS‐pc (Rohlf, 2000).

Different measures of *genetic diversity* were calculated with the R script AflpDat (Ehrich, 2006; http://www.nhm.uio.no/english/research/ncb/aflpdat/) using the statistical software “R” (version 3.0.2; R Developement Core Team, 2008; http://cran.at.r-project.org/): Nei's gene diversity (*H*_E_ *;* Nei, 1973), frequency down‐weighed marker values (Schönswetter & Tribsch, 2005) and the proportion of polymorphic fragments (*PLP*). Genetic diversity was tested for correlation with peripheral location (i.e., geographical longitude) and population size categories using Spearman's rank correlation coefficient in SPSS (ver. 15.0; ©SPSS, Chicago).

An *AFLP outlier analysis* was originally performed using the software DFDIST, adapted for dominant markers (Beaumont & Balding, 2004; Beaumont & Nichols, 1996). However, as our data represent a hierarchically structured setting, we also performed the extended approach proposed by Excoffier, Hofer, and Foll (2009) running ARLEQUIN (version 3.5.1.2; Excoffier & Lischer, 2010; http://cmpg.unibe.ch/software/arlequin35/Arl35Downloads.html). Outlier analyses are based on the assumption that genetic differentiation between populations at certain loci under divergent or balancing selection should be higher or lower than that at neutral loci, respectively. Utilizing a Bayesian approach estimating allelic frequencies for dominant markers, previously developed by Zhivotovsky (1999), the software DFDIST uses simulations to generate a neutral distribution based on an infinite island model, but in ARLEQUIN based on an explicit hierarchical island model. Pairwise regional analyses were performed with the four populations from the Swabian Alb exclave representing the periphery tested against the two more central regions (i.e., Eastern Austria and Central Hungary) as well as the hierarchical approach covering all three regions (only ARLEQUIN results are shown). Running a script written for the R statistical package (ver. 3.1.3; R Core Team, 2015) as implemented in ARLEQUIN, AFLP outlier fragments were visualized in plots of *F*_ST_ against heterozygosity values (see Data S1). In addition, a further enhanced Bayesian analysis (cf. Pérez‐Figueroa, García‐Pereira, Saura, Rolán‐Alvarez, & Caballero, 2010) was performed with the software BAYESCAN (version 2.1; Foll & Gaggiotti, 2008; http://www.cmpg.unibe.ch/software/bayescan/) validating the reliability of the detected outliers. BAYESCAN is based on the multinomial Dirichlet model and assumes that allele frequencies within populations follow such a distribution. To directly deduce the posterior probability of each locus being under selective pressure, this approach defines and compares two alternative models (i.e., one including and one excluding the effect of selection). Model selection is then based on posterior odds (PO), which represent the ratio of posterior probabilities of the models and are used as a threshold for considering a locus to be under selection (Fischer, Foll, Excoffier, & Heckel, 2011; Foll & Gaggiotti, 2008). This analysis was carried out using the default settings for dominant binary AFLP data. Finally, we used the conservative False Discovery Rate (FDR) of 0.01 (i.e., the expected proportion of false positives among all significant results) to control for multiple testing.

#### 2.5.2 cpDNA sequence data

Using TCS (version 1.21, Clement, Posada, & Crandall, 2000; http://w3.ualg.pt/~rcastil/SOFTWARE_WINDOWS/TCS1.21/docs/TCS1.21.html; http://darwin.uvigo.es/software/tcs.html), a program to create statistical parsimony haplotype networks, the alignment of cpDNA sequences was analyzed. For this analysis, insertions/deletions longer than one base pair as well as inversions were coded as single‐step mutations, and sequence gaps were treated as a fifth character state. Genetic diversity indices (i.e., nucleotide [π] and haplotype diversity [*h*]) based on cpDNA variation were again calculated using ARLEQUIN (ver. 3.5.1.2; Excoffier & Lischer, 2010).

## 3 RESULTS

### 3.1 AFLPs

Initially, 288 individuals representing four populations per study region were investigated. Final analyses were then based on 267 individuals (85 from the Swabian Alb region, 91 from Lower Austria, and 91 from Central Hungary), as for 21 individuals the generation of reliable AFLP fragment patterns was not possible. The three primer combinations resulted in 219, 170, and 169 AFLP fragments (mean: 186 fragments, *SD* ± 28.6), respectively. From 558 AFLP fragments, one was monomorphic (0.2%) and therefore excluded from further statistical analyses.

The individual‐based *distance analysis* (i.e., neighbor‐net; see Data S2) basically illustrated the regional structure of geographical origin whereupon individuals mainly clustered according to their population origin within the study regions. Individuals of the Swabian Alb region are more clearly separated from most of the Austrian and Hungarian populations, than Austrian and Hungarian populations from each other. Additionally, a significant isolation‐by‐distance pattern (Mantel *r *=* *0.684; *p* = .0003) across all populations, but not within each of the three study regions (Mantel tests; *p* > .05) was detected.

**Figure 2:**
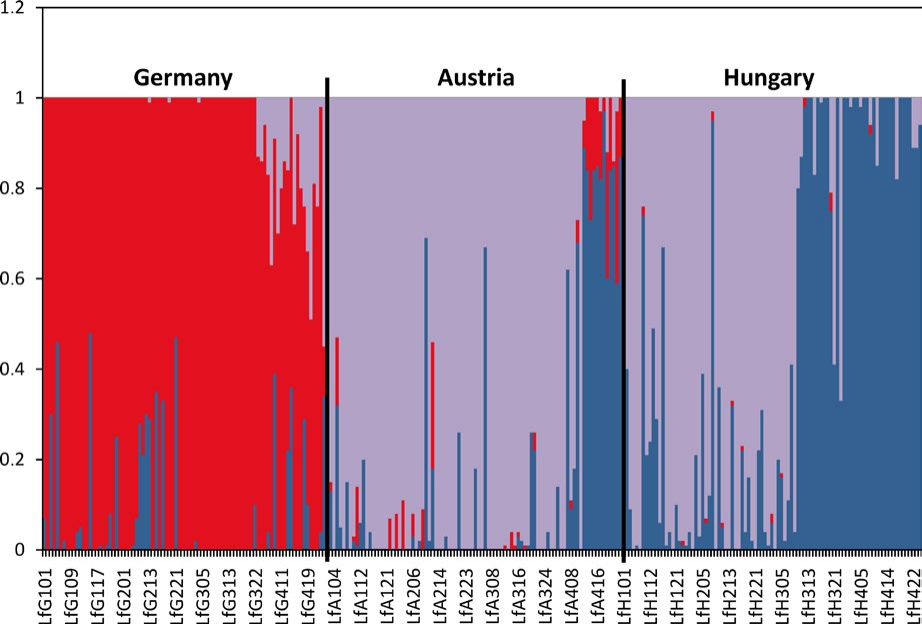
AFLP Bayesian clustering analysis. AFLP admixture analysis of all populations (based on mixture clustering of individuals; *K* = 3; BAPS 3.2.) showing low admixture within the Swabian Alb region but higher amounts of admixture between the other two study regions (i.e., Lower Austria and Central Hungary)

*Bayesian clustering analyses* of population structure revealed an optimal number of *K *=* *3 groups. All populations from the Swabian Alb region were assigned to one group (1, subsequently called the “Swabian group”) while the Austrian and Hungarian populations showed a varying allocation to the remaining two groups: (2) A1, A2, A3, H1, H2; and (3) A4, H3, H4. Moreover, an admixture analysis (based on *K *=* *3; see Figures 1 and 2) resulted in a higher amount of admixture between individuals of the two latter groups, especially among the populations A4, H1 and H3. *AMOVAs* revealed a differentiation of 19% of total variance among all populations (Table 2). Considering the regional origin, a hierarchical AMOVA resulted in a significant differentiation of 12% among regions. A similar result was found when calculations were based on the grouping of the Bayesian analyses (*K *=* *3), leading to a slightly lower regional differentiation of 11.5%; in both cases, the populational differentiation within groups reached about 10%. Population differentiation within each of the three study regions was calculated separately and resulted in the highest differentiation among Hungarian populations (13.4%) and the lowest between Austrian populations (8.6%; Table 2).

**Table 2:** Analyses of molecular variance

Source of variation	d.f.	Sum of squares	Variance components	% Total variance
All populations analyzed				
Among all populations	11	1798.429	6.16690	18.97***
Within populations	255	6718.687	26.34779	81.03
Three groups according to the three geographical regions				
Among groups	2	915.950	4.04165	12.02***
Among populations within groups	9	882.479	3.22755	9.60***
Within populations	255	6718.687	26.34779	78.38***
Three groups according to the results of Bayesian clustering				
Among groups	2	879.557	3.85836	11.48***
Among populations within groups	9	918.872	3.41295	10.15***
Within populations	255	6718.687	26.34779	78.37***
Subset: Isolated populations in the Swabian Alb region				
Among all populations	3	262.802	3.03539	11.54***
Within populations	81	1885.221	23.27434	88.46
Subset: Peripheral populations in the Lower Austrian region				
Among all populations	3	298.929	2.97946	8.55***
Within populations	87	2772.565	31.86857	91.45
Subset: Populations in the more central Central Hungarian region				
Among all populations	3	320.748	3.66469	13.40***
Within populations	87	2060.900	23.68851	86.60

Nonhierarchical and hierarchical analyses of molecular variance (AMOVAs) in the context of the three geographical regions and the groups obtained from Bayesian clustering analysis. Significance levels: *p < .05; **p < .01; ***p < .001.

Running both pairwise and hierarchical *AFLP outlier analysis* using ARLEQUIN revealed twelve AFLP fragments with values higher than the 99% confidence level of the neutral expectation (Table 3; see also Data S1). Running the Bayesian approach as implemented in BAYESCAN by setting FDR = 0.01 (here when PO > 12, i.e., providing decisive evidence for possible selection), revealed 14 outliers, of which three were not detected by ARLEQUIN. Otherwise, one AFLP outlier was just detected by the ARLEQUIN approach. When AFLP fragments with low explanatory power (i.e., possible type I errors, poorly reliable outliers; Bonin, Taberlet, Miaud, & Pompanon, 2006) were discarded (these discarded AFLP fragments include the single outlier only detected by the ARLEQUIN [pairwise] and not by the BAYESCAN approach), seven AFLP fragments remained from both outlier analyses possibly indicating divergent selection (Table 3). However, with respect to the distribution of these AFLP outlier fragments across the three study regions only *one* fragment (no. 256; Table 3) pointed at possible regional adaptation due to divergent selection in the westernmost Swabian Alb exclave. Interestingly, the latter AFLP outlier fragment was previously also detected by using the more simple approach of DFDIST.

**Table 3:** AFLP outlier analysis

Type of AFLP outlier fragments	No. of AFLP outlier fragments observed	Most likely cause
Fragments appearing in one pairwise regional analysis & not in the hierarchical approach	2	False positive (type I error)
Fragments appearing in one pairwise regional analysis & within the hierarchical approach	3	Poorly reliable outlier
Fragments appearing in at least two regional comparisons & in the hierarchical approach	7	Possible regional adaptation
But fragment absent in A	1	
But fragments absent or extremely rare in G	3	
But fragment absent in H	1	
The fragment present only in A	1	
The fragment present only in G (no. 256)	1	… to conditions in the westernmost exclave

Results of the AFLP outlier analysis based on 289 amplified fragment length polymorphism (AFLP) fragments (maximum allowed fragment frequency = 0.98), performed using the ARLEQUIN software at the 99% confidence level. All listed AFLP outlier fragments except one representing the first category (i.e., likely a false positive) were also detected using the Bayesian approach (FDR = 0.01; PO > 12) as implemented in BAYESCAN (see Results for details).

As estimators of *genetic diversity* Nei's gene diversity (*H*_E_) and the proportion of polymorphic markers (*PLP*) revealed similar results for all populations (cf. Table 1). The absolute lowest value was observed in the Hungarian population Üröm (H4; *PLP*=0.27, *H*_E_ = 0.07; cf. Table 1). On regional average, the Austrian region revealed the highest values (mean *H*_E_ = 0.11, *SD* = 0.02; *PLP* = 0.43, *SD* = 0.06) of regional genetic diversity, while for the Swabian Alb (*H*_E_ = 0.08, *SD* = 0.005; *PLP* = 0.31, *SD* = 0.03) and the Hungarian region (*H*_E_ = 0.09, *SD* = 0.01; PLP = 0.33, *SD* = 0.04) analyses indicated lower, but similar genetic diversity. Frequency down‐weighed marker values (from sums) ranged from 51.13 in the population Klingenstein (G4, Swabian Alb) to 151.67 in the population Bisamberg (A3, Lower Austria). On regional scale, the pattern again revealed highest values in Austria (mean DW = 91.19; *SD* = 40.46) and lower genetic diversity in the Swabian Alb (mean DW = 61.71; *SD* = 10.89) and the Hungarian region (DW = 66.44; *SD* = 2.95). None of the genetic diversity estimates was correlated with neither population size nor geographical longitude (i.e., peripherality).

### 3.2 cpDNA sequences

From the 104 originally sequenced individual *L. flavum* samples, 95 could be finally used for further analyses (others removed due to incomplete sequence information). Therefore, number of individual sequence information range between six and nine samples per population (Table 1). The total length of the *rpL16 intron* and the *atpI‐H* sequences was 1,038 bp and 1,177 bp, respectively. In total, the alignment was therefore 2,215 bp long, resulting in 53 different haplotypes. All twelve populations were polymorphic, mainly sharing haplotypes across individuals/populations within a given study region.

**Figure 3:**
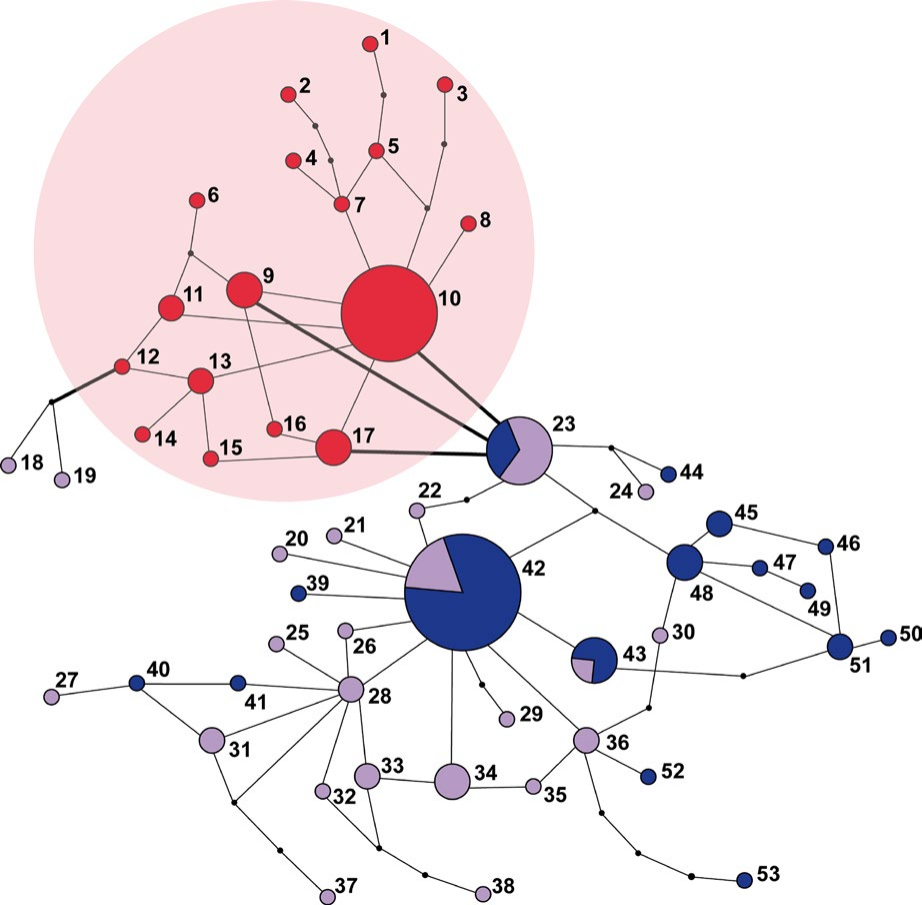
CpDNA haplotype network. TCS haplotype network based on combined cpDNA sequence data. All populations of the Swabian Alb region form together one haplotype group (shaded in red) separated by one specific point mutation (at bp 606 in *rpL16;* bold lines) from the Pannonian haplotype group (including both, Hungarian and Austrian populations)

A *haplotype network* calculated with TCS revealed two regionally defined haplotype groups, in the following called the Swabian haplotype group and the Pannonian haplotype group (Figure 3). The Swabian haplotype group basically included 17 haplotypes consisting of all individuals from the Swabian Alb region, but also two individuals from the Lower Austrian region (haplotypes 18, 19; both from the Eichkogel [A1]). However, there is one specific point mutation (at bp 606 in the *rpL16* intron) differentiating all Swabian Alb samples from the rest (including the latter two Eichkogel samples). In the Pannonian group (i.e., Austrian and Hungarian individuals), consisting of 36 haplotypes, no further obvious regional substructuring was found. Within this Pannonian haplotype group three haplotypes (i.e., 23, 42, and 43) included both, Hungarian and Austrian individuals of two to three different populations (but neither including individuals of the population Höbenbach [A4] nor Belsöbarand [H2], see Data S3).

*Genetic diversity* measures based on cpDNA haplotypes revealed the lowest diversity for the population Üröm [H4], which was basically also the case in the AFLP analyses (Table 1). The highest populational diversity values were found in the Austrian population Perchtoldsdorfer Heide [A2] and the Hungarian population Belsöbarand [H2]. However, at the regional level cpDNA haplotype diversities of the German and the Austrian study regions were similarly high, while the Austrian region showed the highest nucleotide diversity. Therefore, both haplotype and nucleotide diversity values were lowest in the Hungarian region (Table 1). Following the guideline by Grant and Bowen (1998; see also Avise, 2000; Kropf et al., 2006; Gussarova, Alsos, & Brochmann, 2012) the high haplotype and nucleotide diversity in the Austrian study region is to be interpreted as indicating large stable populations with a long‐lasting in situ history, the lower nucleotide diversity combined with an high haplotype diversity in the Swabian Alb as indicating population growth after an initial population bottleneck allowing for accumulation of *de novo* mutations, while low nucleotide and haplotype diversity in the Hungarian study region is likely demonstrating a recent population bottleneck.

## 4 DISCUSSION

### 4.1 Genetic variation and diversity along the study transect

Peripheral populations of widely distributed species often occur in lower densities, especially in particular habitats, are smaller in population size, and therefore are isolated and genetically more differentiated among each other (e.g., Eckert et al., 2008; Sagarin & Gaines, 2002; Wagner, Durka, & Hensen, 2011). Hence, biogeographical hypotheses expect decreasing genetic diversity and possibly reduced fitness toward the range edge (Eckert et al., 2008; Sagarin & Gaines, 2002; Sexton et al., 2009). A recent review of Abeli et al. (2014), focusing on demographic traits of peripheral populations, elucidated that most studies do not support those general assumptions. Moreover, Pironon, Villellas, Morris, Doak, and García (2015) re‐evaluated the “center‐periphery hypothesis” by using different approaches of centrality and marginality: the species current *geographical* distribution, the *climatic* optimum and the *historic* distribution at the LGM. Our peripheral study transect, however, represents the current geographical distribution at the species’ absolute range limit in Central Europe (cf. Figure 1), at which the number of phylogeographic studies on steppe plants is limited (cf. Kajtoch et al., 2016).

However, regarding the hypothesized periphery gradients of population sizes/densities, we estimated lowest population sizes (average of the respective populations) in the Austrian and highest in the Swabian Alb region, that is., not following the so‐called abundant center distribution (Sagarin & Gaines, 2002). Otherwise, the total number of existing populations known from the respective study regions was indeed by far the lowest in the Swabian Alb region (with only six current populations known; Wörz, Hölzer, & Thiv, 2015; but see also Demuth et al., 1992). Therefore, total (regional) abundance of *L. flavum* along the study transect is indeed following the prediction of the abundant center distribution (Sagarin & Gaines, 2002). Nevertheless, in our AMOVAs *L. flavum* populations did not show an increasing population differentiation (within regions) in a westward direction. The highest differentiation among populations within a study region was found in Hungary while the differentiation among the Swabian Alb populations was only slightly lower. Moreover, no clear evidence for reduced genetic diversity within the absolute westernmost exclave could be observed in both, the mainly nuclear AFLP and the chloroplast DNA data. Actually, the estimated diversity values were more similar for the Swabian Alb and the Hungarian populations although the latter ones represent the more continuous part of the distribution range. This result corresponds well with findings by Kajtoch et al. (2016), who found high intraregional genetic diversity mainly at the species range edges and moderate‐to‐high interregional distinctiveness. The absolute lowest genetic diversity values found in the Hungarian population Üröm (Table 1) might specifically indicate locally suboptimal conditions (i.e., representing a small area increasingly covered by *Pinus nigra*) and a *recent* population decline, there. In summary, although most assumptions of the abundance center distribution hypothesis (Sagarin & Gaines, 2002) are valid in our peripheral *L. flavum* study section, all genetic parameters analyzed did not follow expectations probably due to a strong species‐specific historic component (see next chapter; cf. Pironon et al., 2015).

### 4.2 Relict status of the Swabian Alb *Linum flavum* populations

During glacial stages of the Quaternary period cold continental steppes dominated vast areas of Central Europe (cf. Lang, 1994). However, it remains open if more thermophilic steppe elements also occurred within these areas locally, and if steppe elements could have persisted the LGM in situ, as has been argued for woody plant species by Stewart and Lister (2001, but see also Stewart, Lister, Barnes, & Dalén, 2010), and also for thermophilic animal species by Schmitt and Varga (2012).

Nevertheless, climatic oscillations during the Quaternary period enabled steppic grassland species to expand their ranges from eastern and southeastern Europe in a westward direction along the mountainsides of the Carpathians and the Lower Danube Corridor to Central Europe (Litzelmann, 1938; Magyari et al., 2010). Such large‐scale expansions took certainly place in several waves during early postglacial times: first probably between 11,000 and 10,250 cal year BP (Magyari et al., 2010), followed by broad‐leaf forests mainly expanding from their main southern refugia between 9,900 and 5,000 ya, where steppelike grassland species could have persisted in small open areas (Feurdean et al., 2012, 2015); then again expanding their range during anthropogenic influenced intensive deforestations in the Late Holocene (Cieślak, 2014; Feurdean et al., 2015; Magyari et al., 2010, 2012; Soó, 1959). As a consequence, there had been phases of a far more westward extension of the steppe zone into Central and Southwestern Europe, and nowadays Central European occurrences of steppe species have subsequently become disjunct and situated outside the actual steppe zone (Bredenkamp et al., 2002; Cieślak, 2014; Soó, 1959).

While it still remains uncertain, in which of the early above‐mentioned expansion phase *L. flavum* reached southern Germany or even survived the LGM there, we definitely can exclude a scenario of human‐mediated expansion in the late Holocene. Given the genetic distinctiveness of the Swabian Alb populations combined with the comparatively high genetic diversity observed, we have to assume a long‐term persistence within this region (i.e., assuming an arrival during the Pleistocene) rather than a postglacial arrival (see also Cieślak, 2014; Kajtoch et al., 2016; Schmitt & Varga, 2012). Moreover, as the Swabian Alb, located between the Upper Rhine in the southwest and the Nördlinger Ries in the northeast, remained unglaciated during the LGM (Wilmanns, 2005), habitat conditions might have effectively allowed long‐term persistence and survival, there.

*Linum flavum* is represented by very few populations at its absolute westernmost distribution limit in the Swabian Alb region in southern Germany. Beside two additional nonsampled locations, the four investigated populations from the Swabian Alb region (i.e., three populations from BW and one from the Iller river valley in Bavaria) are currently the only documented populations in this region (Wörz et al., 2015). There are older records of *L. flavum* known from southern and southeastern Bavaria (Bettinger et al., 2013), but nowadays these populations must be considered as extinct. The former distribution of *L. flavum* in the German region indicates a previously more continuous occurrence along the Danube valley and possibly give evidence for a western direction of expansion likely following the Danube valley, originating from the Pannonian part of the species’ distribution range (cf. Litzelmann, 1938). Bavarian populations, located along the north–south‐oriented river valleys of the Iller (incl. one still existing population) and the Lech (extinct), may have expanded locally during previous, climatically more suitable times from the Danube toward the south (Bettinger et al., 2013).

Interestingly, the habitats populated by *L. flavum* in this traditional cultural Swabian Alb landscape are more mesic (Demuth et al., 1992; own obs.) compared to the habitats in the Pannonian region. Furthermore, those populations occur—within our study transect—at the highest altitudes (>500 m a.s.l.; Table 1) indicating, for instance, less summer‐hot conditions. Therefore, these slightly differing site conditions possibly indicate specific (i.e., marginal) habitat requirements, which *L. flavum* obviously overcomes due to its ecological breadth and probably not by local adaptation as suggested by our AFLP outlier analysis (see Table 3).

In this context, our AFLP analyses revealed a high degree of genetic distinctiveness of all Swabian Alb populations (Figure 2) and a higher amount of mixture between populations of the Pannonian (i.e., Austrian and Hungarian) region (see also Cieślak, 2014). This pattern was basically also present within the cpDNA sequence variation, where all Swabian Alb individuals were differentiated from the Pannonian ones by a specific point mutation (Figure 3). Interestingly, this distinctiveness is not accompanied by low genetic diversity; rather, the AFLP and cpDNA analyses point at comparatively high levels of overall genetic diversity (as has been compiled for other steppe plants also by Kajtoch et al., 2016). Furthermore, high cpDNA haplotype diversity can be interpreted as specific genetic diversity component indicating long‐term persistence in situ, that is, allowing for *de novo* generation of genetic variation after early arrival in this region (Grant & Bowen, 1998; Avise, 2000; see also Kropf et al., 2006; Gussarova et al., 2012). Given sufficient time, genetic diversity will evolve in an once colonized area as a result of the accumulation of *de novo* mutations postdating population separation, and/or effects of lineage sorting from a highly polymorphic ancestral gene pool (Avise, 2000). The observed genetic structuring (incl. a significant isolation‐by‐distance pattern across regions) and the comparatively high genetic diversity values (especially regarding cpDNA haplotype numbers and diversity) therefore demonstrate long‐standing biogeographical vicariance, perhaps indicating “cryptic refugia” or extra‐Mediterranean refugia as discussed by Stewart and Lister (2001) and Schmitt and Varga (2012). In this sense, our results provide evidence of the relict status of the nowadays highly isolated Swabian Alb populations.

Considering a phylogeographic pattern of successive vicariance (cf. Kropf et al., 2006), the possibly *earlier* isolation of the Swabian Alb exclave and partly differing habitat conditions, a process of local adaptation might provide an additional possible explanation for the (genetic) distinctiveness of these populations. In this respect, we have to note that genetic markers analyzed here are mainly not suitable for the indication of selective processes. However, at least our AFLP outlier analyses (cf. Table 3) provide only little, if any conclusive evidence of local divergent selection within the Swabian Alb study region.

## 5 CONCLUSIONS ON CONSERVATION GENETIC ASPECTS

Although the conservation need of peripheral (plant) populations is controversially assessed (Leppig & White, 2006; Lesica & Allendorf, 1995), to our opinion the conservation value of the Swabian Alb populations is unquestionable. Beside their current Red List status and the declining population sizes/densities in this area (Bettinger et al., 2013; Demuth et al., 1992; Király, 2007; Korneck et al., 1996; Niklfeld & Schratt‐Ehrendorfer, 1999), their genetically distinctiveness and comparatively high genetic diversity highlight the urgent need for preserving this specific gene pool (Kajtoch et al., 2016; Leppig & White, 2006; Lesica & Allendorf, 1995; Schmitt & Varga, 2012).

In the context of genetic fraction and uniqueness (Petit, El Mousadik, & Pons, 1998) of our investigated peripheral populations, we observed that 89 (16.0%) of 557 polymorphic AFLP fragments were only present in the Swabian Alb exclave. This value is in‐between the other two regional values (i.e., 110 private AFLP fragments in Eastern Austria [= 19.7%]; 77 fragments in Central Hungary [13.8%]) and therefore represents *one‐third* of all 276 regionally unique AFLP fragments. Moreover, we found altogether 53 cpDNA haplotypes of which 17 were exclusively observed in the Swabian Alb study region (i.e., 30%). As regionally proportions of intraspecific genetic diversity are only rarely quantitatively analyzed (Pérez‐Collazos, Segarra‐Moragues, & Catalán, 2008; but see Bálint et al., 2011 for mountain stream insects), directly comparable data for other steppe taxa are not available.

However, Kajtoch et al. (2016) found partially low‐to‐moderate intraregional genetic diversity within the continuous part of Pannonian (steppe) species’ ranges, but considerably higher values for populations occurring in isolated habitats at the absolute distribution limit (e.g., in Poland or Germany; cf. *Scorzonera purpurea*,* Serratula lycopifolia*, and *Stipa pennata*). Nevertheless, other investigations covering the intraspecific genetic constitution of steppe plants indicate contrary to our *L. flavum* study mainly reduced genetic diversity in Central Europe, that is, also reduced uniqueness (e.g., *Astragalus exscapus* Becker, 2003; *Stipa capillata* Hensen et al., 2010; *Stipa pulcherrima* Durka et al., 2013). Although we have to treat our quantitative genetic estimates with caution, as we have not sampled total genetic variation throughout the vast distribution range of *L. flavum*, the result of a distinctive and diverse Swabian Alb gene pool is likely to prove true even when extending the sampling (cf. Cieślak, 2014; Kajtoch et al., 2016). Nevertheless, the estimated high genetic diversity and uniqueness is confronted by decreasing population sizes and a low total number of populations in the Swabian Alb region. Therefore, we also have to consider a possible time lag (cf. Lindborg & Eriksson, 2004) between population (size) decline and a (consequently) loss of genetic diversity (see also Kropf, 2012) in that region.

## CONFLICT OF INTEREST

None declared.

## DATA ACCESSIBILITY

European Nucleotide Archive (ENA) accession numbers for DNA sequences: *rpL16* intron (LT799944‐65) and *atpI‐H* (LT827070‐103).

## AUTHOR CONTRIBUTIONS

M.K. and K.P. conceived the idea, interpreted the data, and wrote most of the manuscript; K.P., K.B., and M.K. analyzed the data, and K.B. wrote part of the manuscript and prepared the map. K.P. and K.B. performed the molecular work, and M.K., K.B., M.H., and M.T. collected the plant material. M.H., M.T., and K.B. critically revised the manuscript mainly drafted by K.P. and M.K. All authors finally approved the manuscript.
