# Supplementary figures and images for: No obvious genetic erosion, but evident relict status at the westernmost range edge of the Pontic‐Pannonian steppe plant Linum flavum L. (Linaceae) in Central Europe
Source: Ecol Evol. 2017 Jul 14;7(16):6527–39. doi: 10.1002/ece3.2990 (PMC5574788; doi:10.1002/ece3.2990)

Supporting Information 1. **AFLP outlier analyses based on regional comparisons.**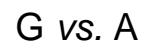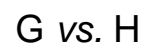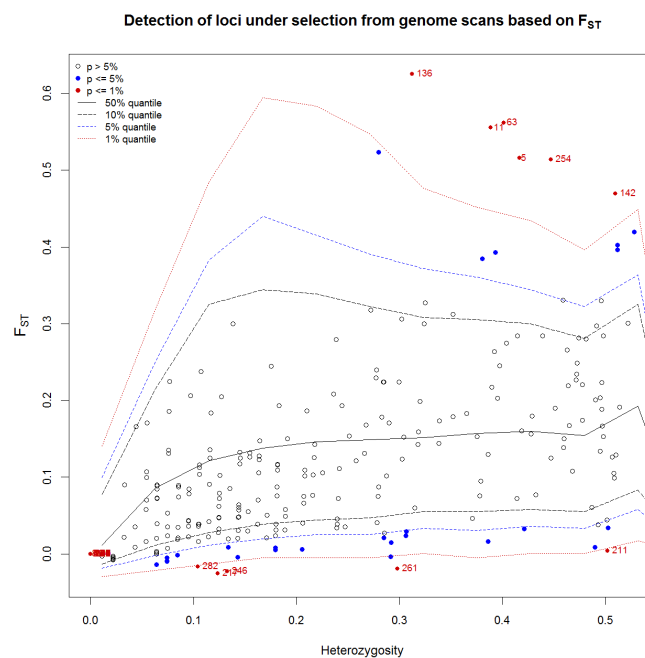

Supplement: Supplementary file 1 [file ECE3-7-6527-s001.pdf]

Supporting Information 2. **Individual-based Neighbour-Net of *Linum flavum*.**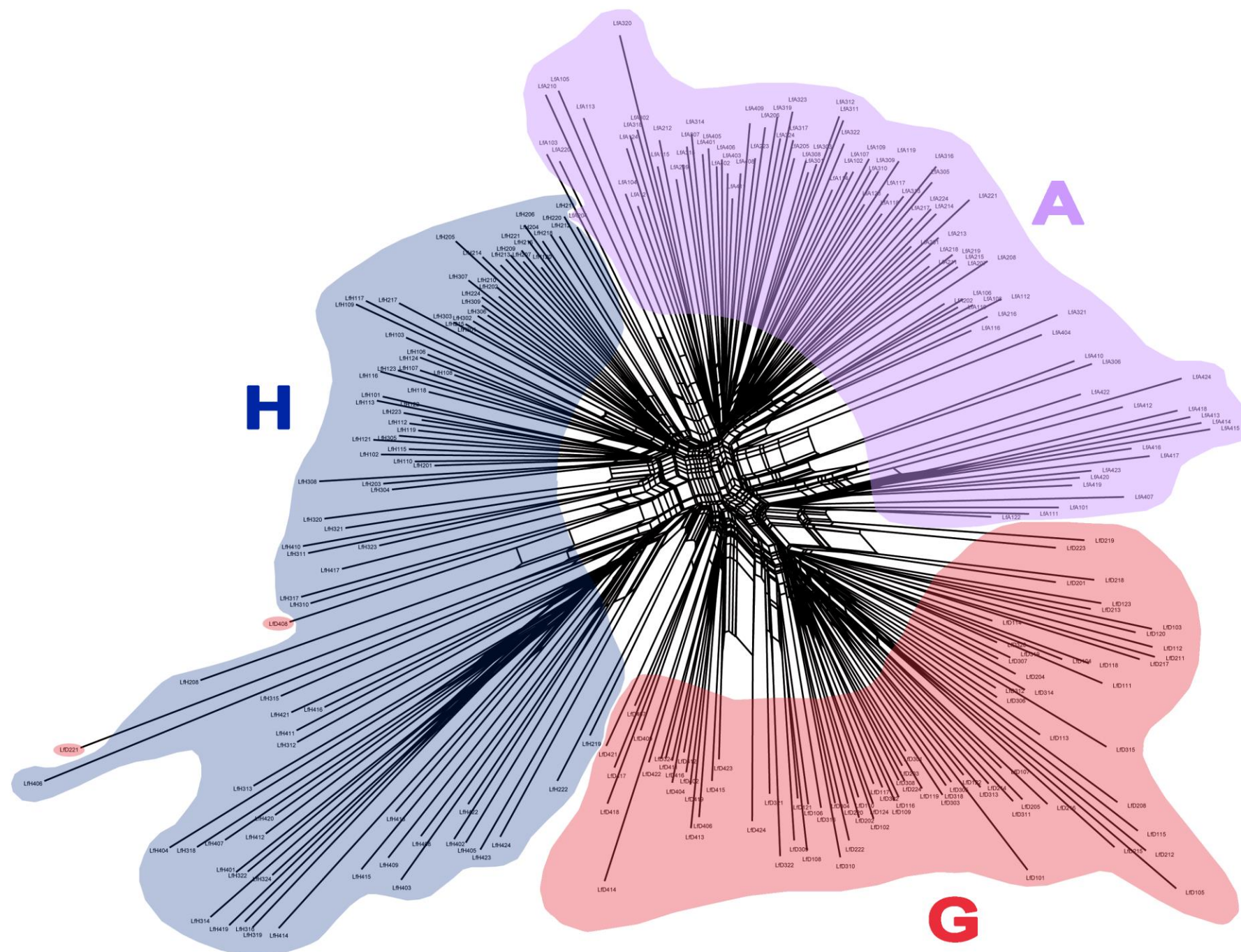

Supplement: Supplementary file 2 [file ECE3-7-6527-s002.pdf]
