# Supplementary material for: No obvious genetic erosion, but evident relict status at the westernmost range edge of the Pontic‐Pannonian steppe plant Linum flavum L. (Linaceae) in Central Europe
Source: Ecol Evol. 2017 Jul 14;7(16):6527–39. doi: 10.1002/ece3.2990 (PMC5574788; doi:10.1002/ece3.2990)
Supplement: Supplementary file 3 [file ECE3-7-6527-s003.pdf]

Supporting Information 3. **Distribution of cpDNA haplotypes across populations and regions.**

| **Haplo-** | | **G** | | | | **A** | | | | **H** | | | **sum** |
|---|---|---|---|---|---|---|---|---|---|---|---|---|---|
| **type** | LfG1 | LfG2 | LfG3 | LfG4 | LfA1 | LfA2 | LfA3 | LfA4 | LfH1 | LfH2 | LfH3 | LfH4 | |
| 1 | | 1 | | | | | | | | | | | 1 |
| 2 | | | 1 | | | | | | | | | | 1 |
| 3 | | | | 1 | | | | | | | | | 1 |
| 4 | 1 | | | | | | | | | | | | 1 |
| 5 | | 1 | | | | | | | | | | | 1 |
| 6 | | 1 | | | | | | | | | | | 1 |
| 7 | | 1 | | | | | | | | | | | 1 |
| 8 | | | 1 | | | | | | | | | | 1 |
| 9 | | 2 | 1 | | | | | | | | | | 3 |
| 10 | 2 | | 3 | 4 | | | | | | | | | 9 |
| 11 | | 1 | 1 | | | | | | | | | | 2 |
| 12 | 1 | | | | | | | | | | | | 1 |
| 13 | | | | 2 | | | | | | | | | 2 |
| 14 | | | | 1 | | | | | | | | | 1 |
| 15 | 1 | | | | | | | | | | | | 1 |
| 16 | | | 1 | | | | | | | | | | 1 |
| 17 | 1 | 1 | 1 | | | | | | | | | | 3 |
| 18 | | | | | 1 | | | | | | | | 1 |
| 19 | | | | | 1 | | | | | | | | 1 |
| 20 | | | | | | 1 | | | | | | | 1 |
| 21 | | | | | 1 | | | | | | | | 1 |
| 22 | | | | | | | 1 | | | | | | 1 |
| 23 | | | | | 4 | | | | | | **2** | | 6 |
| 24 | | | | | 1 | | | | | | | | 1 |
| 25 | | | | | | 1 | | | | | | | 1 |
| 26 | | | | | | 1 | | | | | | | 1 |
| 27 | | | | | | 1 | | | | | | | 1 |
| 28 | | | | | | | 2 | | | | | | 2 |
| 29 | | | | | | | 1 | | | | | | 1 |
| 30 | | | | | 1 | | | | | | | | 1 |
| 31 | | | | | | | 2 | | | | | | 2 |
| 32 | | | | | | 1 | | | | | | | 1 |
| 33 | | | | | | | | 2 | | | | | 2 |
| 34 | | | | | | | | 3 | | | | | 3 |
| 35 | | | | | | | | 1 | | | | | 1 |
| 36 | | | | | | 1 | 1 | | | | | | 2 |
| 37 | | | | | | 1 | | | | | | | 1 |
| 38 | | | | | | | | 1 | | | | | 1 |
| 39 | | | | | | | | | 1 | | | | 1 |
| 40 | | | | | | | | | | 1 | | | 1 |
| 41 | | | | | | | | | 1 | | | | 1 |
| 42 | | | | | | | **2** | | 5 | | | 4 | 11 |
| 43 | | | | | | **1** | | | 1 | | | 2 | 4 |
| 44 | | | | | | | | | | | 1 | | 1 |
| 45 | | | | | | | | | | | 2 | | 2 |
| 46 | | | | | | | | | | 1 | | | 1 |
| 47 | | | | | | | | | | 1 | | | 1 |
| 48 | | | | | | | | | | 2 | 1 | | 3 |
| 49 | | | | | | | | | | 1 | | | 1 |
| 50 | | | | | | | | | | 1 | | | 1 |
| 51 | | | | | | | | | | | 2 | | 2 |
| 52 | | | | | | | | | | 1 | | | 1 |
| 53 | | | | | | | | | 1 | | | | 1 |
| **sum** | 6 | 8 | 9 | 8 | 9 | 8 | 9 | 7 | 9 | 8 | 8 | 6 | |
